# A new two-strip TLC method for the quality control of technetium-99m mercaptoacetyl-triglycine (^99m^Tc-MAG3)

**DOI:** 10.1186/s41181-018-0040-5

**Published:** 2018-03-14

**Authors:** Marietta Straub, Michel Leresche, Claude Pilloud, Fabien Devynck, Nicolas Stritt, Rolf Hesselmann

**Affiliations:** 1 Institute of Radiation Physics, Lausanne University Hospital, Lausanne, Switzerland; 2 Federal Office of Public Health, Bern, Switzerland

**Keywords:** Radiopharmaceutical Quality Control, ^99m^Tc-MAG3, Radiochemical Purity, Thin Layer Chromatography

## Abstract

**Background:**

^99m^Tc-mercaptoacetyl-triglycine (^99m^Tc-MAG3) has been used for dynamic renal imaging since about 30 years. Free pertechnetate (^99m^TcO_4_), colloidal ^99m^Tc ((^99m^TcO_2_)_n_), ^99m^Tc-tartrate (precursor), precomplexes (^99m^Tc-(MAG3)_x_) and lipophilic ^99m^Tc-MAG2 are the main radiochemical impurities that may occur in the preparation. The total amount of these impurities has to be identified before release of the product for patient administration to guarantee patient safety and good image quality. The European Pharmacopoeia suggests a method based on high-pressure liquid chromatography analysis in combination with a paper chromatography. This analytical method is time consuming, expensive and requires specially trained technicians. As a consequence, it is not widely applied in nuclear medicine radiopharmacies.

**Results:**

We developed a simple method for radiochemical purity testing of ^99m^Tc-MAG3. The method is based on thin layer chromatography with two strips to be developed in parallel. Method validation was carried out in comparison to the official methods of the companies and to the European Pharmacopoeia method. It was tested on specificity, accuracy, robustness and precision.

**Conclusion:**

The proposed method is able to identify and quantify the sum of all impurities occurring in the preparation, respecting the acceptance criteria for the radiochemical purity defined by the official methods. Hydrophilic and lipophilic compounds are identified separately and results are obtained within less than 20 minutes. Our method is simple, cost effective, fast and is suitable for employing dose calibrators or radiometric scanners.

**Electronic supplementary material:**

The online version of this article (10.1186/s41181-018-0040-5) contains supplementary material, which is available to authorized users.

## Background

In the early 80’s, ^99m^Tc-MAG3 was introduced as a new dynamic renal imaging agent (e.g. (Fritzberg et al., 1986; Taylor et al., 1987; Taylor et al., 1987; Brandau et al., 1988; Bormann et al., 1995)). Today, the preparation is commonly applied in nuclear medicine. For the preparation of ^99m^Tc-MAG3 commercial sterile kits are available in form of a lyophilized pharmaceutical product. The labeled ^99m^Tc-MAG3 is obtained by adding sodium pertechnetate from a ^99m^Tc radionuclide generator (e.g. (van Hemert et al., 2005; Nosco et al., 1993; Seetharaman et al., 2006; Chen et al., 1993; Package insert for Technescan MAG3. Mallinckrodt Suisse SA, 2003; Package insert for ROTOP-MAG-3 Kit. Heider AG, 2006; Package insert for Technescan MAG3. Mallinckrodt Suisse SA, 2015)). Specific biodistribution of ^99m^Tc-MAG3 is crucial for the patient safety and image quality and the radiochemical purity (RCP) of the radiopharmaceutical has to be guaranteed (Fritzberg et al., 1986; Brandau et al., 1988). Radiochemical impurities may lead to imprecise interpretation of diagnostics and can cause unnecessary patient and operator irradiation if the examination has to be repeated. Therefore, a liable quality control (QC) method quantifying the radiochemical impurities occurring in the radiopharmaceutical preparation is indispensable to release the product for patient administration.

**Fig. 1:**
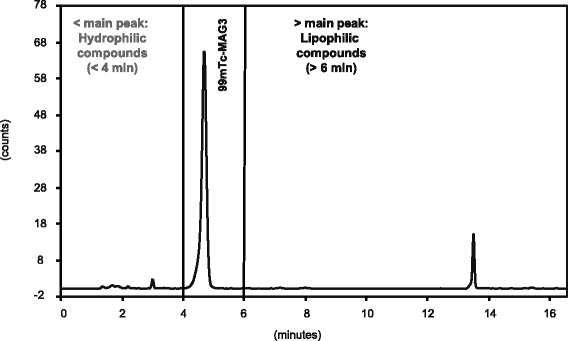
Example chromatogram of Ph.Eur. HPLC analysis of ^99m^Tc-MAG3. Hydrophilic compounds occur < 4.0 minutes, ^99m^Tc-MAG3 is identified at about 4.5 minutes, lipophilic compounds are attributed to the region > 6.0 minute. ^99m^Tc-MAG2 and ^99m^TcO_4_ are the main impurities. ^99m^Tc-tartrate elutes as the first peak followed by small peaks of precomplexes

Potentially occurring radiochemical impurities in the preparation of ^99m^Tc-MAG3 are free pertechnetate (^99m^TcO_4_), colloidal technetium ((^99m^TcO_2_)_n_), ^99m^Tc-tartrate (precursor), precomplexes (^99m^Tc-(MAG3)_x_) and ^99m^Tc-MAG2 (van Hemert et al., 2005; Nosco et al., 1993; Seetharaman et al., 2006; Chen et al., 1993; Package insert for Technescan MAG3. Mallinckrodt Suisse SA, 2003; Package insert for ROTOP-MAG-3 Kit. Heider AG, 2006; Package insert for Technescan MAG3. Mallinckrodt Suisse SA, 2015). The European Pharmacopoeia (Ph.Eur.) method for the quality control of the ^99m^Tc-MAG3 preparation separates and quantifies the hydrophilic components (^99m^Tc-tartrate, ^99m^TcO_4_, ^99m^Tc-(MAG3)_x_, ^99m^Tc-MAG3) and the lipophilic components (mainly ^99m^Tc-MAG2) by high-pressure liquid chromatography (HPLC) analysis (European Pharmacopoeia Edition 7.0, 2008). (^99m^TcO_2_)_n_ is analyzed separately by paper chromatography (PC) likewise described in the Ph.Eur. Fig. 1 gives an overview of an HPLC analysis performed as described in the Ph.Eur. Hydrophilic (< 4 min) and lipophilic (> 6 min) components are separated from ^99m^Tc-MAG3 (main peak) and can be easily quantified to obtain the RCP.

HPCL analysis is not optimal for quality control of radiopharmaceutical preparations in clinical settings, as the necessary infrastructure is usually not available in nuclear medicine departments due to high costs and space limitations. Easy and fast methods based on thin layer (TLC) or paper chromatography are preferable. As a result, regardless of its accuracy, the Ph.Eur. method for the quality control of ^99m^Tc-MAG3 preparations is not widely applied in nuclear medicine centers.

To overcome this problem in daily practice, an alternative method for RCP testing based on solid-phase extraction (SPE method with SEP-PAK C18 columns) was developed by the company and accepted by the Swiss authorities for the quality control (Package insert for Technescan MAG3. Mallinckrodt Suisse SA, 2003). Interestingly, we found disagreement between the suggested method in Switzerland compared to other countries. The by the corresponding competent authorities accepted methods differ between the US, the EU and Switzerland and the acceptance criteria deviate from the specifications defined by the Ph.Eur. Table 1 and 2 summarize the different methods proposed in different countries for the two existing formulations of MAG3, Mallinckrodt Technescan MAG3 (table 1) and MAG3 from ROTOP MAG3 kit (table 2). The total amount of the RCP value varies importantly. TLC and SPE methods accept lower RCP values than the RCP based on the Ph.Eur.

**Table 1:** RPC testing methods for Mallinckrodt Technescan MAG3 preparations available in Switzerland, European countries, the US (Package insert for Technescan MAG3. Mallinckrodt Suisse SA, 2003; Package insert for Technescan MAG3. Mallinckrodt Suisse SA, 2015; European Pharmacopoeia Edition 7.0, 2008; Package insert for Technescan MAG3, 2015; Package insert for Technescan MAG3, 2015) in comparison to the method of the Ph.Eur. (European Pharmacopoeia Edition 7.0, 2008). Each SPC method requires detection of the sum of liphophilic and hydrophilic impurities

	Ph.Eur.	Mallinckrodt Technescan MAG3™ Kit for the Preparation of Technetium Tc 99m Mertiatide, DRN 4334						
Version	01/2008:1372(European Pharmacopoeia Edition 7.0, 2008)	CH 10.2003 (Package insert for Technescan MAG3. Mallinckrodt Suisse SA, 2003)		CH 12.2015 (Package insert for Technescan MAG3. Mallinckrodt Suisse SA, 2015)		US A096I0 R10/2015 (Package insert for Technescan MAG3, 2015)	The Netherlands 10.2015 (Package insert for Technescan MAG3, 2015)	
Method	HPLC + PC	HPLC	Sep-Pak C18	HPLC	Sep-Pak C18	Sep-Pak C18 only	HPLC	Sep-Pak C18
	[%]	[%]	[%]	[%]	[%]	[%]	[%]	[%]
Tc-Mag3 Dt:0	≥ 94	≥ 96	≥ 90	≥ 95	≥ 94	≥ 90	≥ 95	≥ 90
hydrophilic impurities	≤3	% not def.	≤ 5		≤ 3	% not def.	≤ 3	≤ 5
Lipophilic impurities	≤4	% not def.	≤ 5		≤ 4 *	Not measured	≤ 4	≤ 5
Colloïd.Tc-99m (non-elutable imp.)	≤2 (PC)	Not measured	Not measured	Not measured	Not measured	% not def.	Not measured	Not measured

*Lipophilic impurities are attributed to SEP-PAK cartride activity. Normally, at the end the cartridge contains only non-elutable impurities.

**Table 2:** RPC testing methods for ROTOP MAG3 kit preparations available in Switzerland, European countries, the US (Package insert for ROTOP-MAG-3 Kit. Heider AG, 2006; Package insert for ROTOP-MAG-3 Kit, n.d.-a; Package insert for ROTOP-MAG-3 Kit, n.d.-b) in comparison to the method of the Ph.Eur. (European Pharmacopoeia Edition 7.0, 2008). Each method requires detection of the sum of liphophilic and hydrophilic impurities, except for the Rotop method from 2016 (only available for EU countries) where detection of colloidal ^99m^Tc ((^99m^TcO_2_)_n_) as an individual impurity is specified and acceptance criteria are set separately

	Ph.Eur.			MAG3 Kit Rotop			
Version	01/2008:1372 Corrected 7.0 [13]	CH 06.2006 (Package insert for ROTOP-MAG-3 Kit. Heider AG, 2006)		rotop-pharmaka.de SmPC-MAG3-HK-eng-01 [16]		Eu countries 2016 (Package insert for ROTOP-MAG-3 Kit, n.d.-b)	
Method	HPLC + PC	HPLC	Sep-Pak C18	HPLC + PC	Sep-Pak C18	HPLC	TLC
	[%]	[%]	[%]	[%]	[%]	[%]	[%]
Tc-Mag3 Dt:0	≥ 94		≥ 94	≥ 94	≥ 94		≥ 94
hydrophilic impurities	≤3	According to Ph.Eur.	% not def.	≤ 3	% not def.	According to Ph.Eur.	≤ 5 **
Lipophilic impurities	≤4		Not measured	≤ 4	% not def.*		Not measured
Colloïd.Tc-99m (non-elutable imp.)	≤2 (PC)		% not def.	Not measured	Not measured		≤ 2

*Lipophilic impurities are attributed to the SEP-PAK cartridge activity.

**Refers to ^99m^Tc-perchtechnetate in the SPC

The Swiss method of Mallinckrodt Swiss SA, in force today (Package insert for Technescan MAG3. Mallinckrodt Suisse SA, 2015), employs SEP-PAK C18 cartridges to separate hydrophilic and lipophilic radiochemical impurities. The impurities are quantified employing a dose calibrator. As accepted by the local authorities, the SPE method is a valid alternative to the Ph.Eur. method.

Intended to be more user friendly, this method is subject to multiple concerns. Testing its accuracy in a clinical setting, it was found that the RCP was significantly underestimated, which could result in false negative results (Murray et al., 2000). This does not only imply inconvenience for the patient and additional costs for the hospital, it also causes unnecessary radiation exposure of the operators when repeating the labeling and the QC. Handling the syringe and cartridges results in a close contact to the radioactive solution for several minutes, this may increase extremity doses. Additionally, results for RCP using SEP-PAK cartridge based methods were found to be operator dependent due to the influence of unavoidable variations in elution velocity (Vinberg, 2000; Ponto, 2005; Millar & Hesslewood, 2004). This may be the reason for the reported differences in accuracy found in clinical settings versus validation studies by the kit manufacturers.

Based on these drawbacks with the simplified methods, other techniques coming back to TLC systems have been suggested by different groups (e.g. (van Hemert et al., 2005; Nosco et al., 1993; Seetharaman et al., 2006; Chen et al., 1993)). Unfortunately, so far no ideal results being comparable to specifications of the official methods or the Ph.Eur. method, including evaluation of all possible impurities could be achieved. Some of the proposed QC methods are even showing contradictory results (Seetharaman et al., 2006; Chen et al., 1993).

The two goals of our study were to test the Swiss method in force proposed by the company and to develop a compliant TLC method easier to perform than the  SPE method. With this new method we want to guarantee identification of all possible impurities as detected by the Ph.Eur. HPLC and PC method.

Therefore, in a first study, we tested the hypothesis of RCP variations with variations in elution velocity (Vinberg, 2000; Ponto, 2005; Millar & Hesslewood, 2004) and we tested the Swiss SPC in force at the time of the study.

In the second part of our study, we developed and validated a new two-strip TLC method, which is fast, easy to perform, operator independent and applicable using a dose calibrator or TLC scanner, standard equipment of a nuclear medicine department. The developed method does not require HPLC analysis. Radiopharmaceuticals which are prepared from authorized kits and generators according to the instructions of the summary of product characteristics (SPC) are not required to be tested according to the respective pharmacopoeia monograph. Instead, the quality control methods described in the SPC are valid, since they are accepted by the pharmaceutical authority. Our method was validated based on these criteria. Our new TLC method evolves from a TLC system suggested by Chen et al. (Chen et al., 1993) but uses ethanol with 10% water as second solvent. We identified characteristics of all possible impurities by performing individual specificity tests and compared the results with the Ph.Eur. HPLC and PC measurements.

Different to other studies (Seetharaman et al., 2006; Chen et al., 1993), our specificity tests allowed us to identify ^99m^Tc-MAG2 as a possible impurity in the ^99m^Tc-MAG3 formulation. In addition, our method permits separation of ^99m^Tc-MAG3 from ^99m^Tc-tartrate. The method was validated in comparison to the official method in force suggested by the company and shows good agreement with the RPC obtained by the Ph.Eur. method. This allows suggesting this method as the standard RCP testing method in any country. The time required to perform the QC is less than 20 minutes, which responds well to the needs and time constraints in a small scale radiopharmacy of a nuclear medicine imaging department.

## Methods

^99m^Tc-MAG3 was prepared from Technescan MAG3 kits and an Ultra-Technekow Mo-99/Tc-99 Generator from Mallinckrodt according to the labeling procedure version 2003 (Package insert for Technescan MAG3. Mallinckrodt Suisse SA, 2003). For the quality control procedures, ACS grade chemicals, iTLC-SG chromatography strips from Agilent Technologies and highly purified water were used.

### SPE Method

In the first part of this study, liability of the SPC method in force at the time of the study, applying SEP-PAK C18 cartridges (Package insert for Technescan MAG3. Mallinckrodt Suisse SA, 2003), was tested. SEP-PAK cartriges used were SEP-PAK C18 Plus Short Cartridge, 360 mg Sorbent per Cartridge, 55-105 μm Particle Size, 50/pk (WATERS Part.Number WAT020515).

**Fig. 2:**
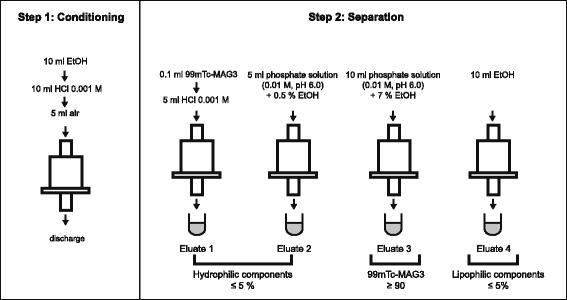
Illustration of Mallinckrodt Pharmaceuticals QC procedure for MAG3 kit (version 2003)

Referring to earlier studies (Chen et al., 1993; Vinberg, 2000; Ponto, 2005), we tested the robustness of the method at different elution rates of 1.7, 10, 20 and 30 ml/min (no indication for an exact elution rate is mentioned in the SPC (Package insert for Technescan MAG3. Mallinckrodt Suisse SA, 2003)). Results were compared with results obtained by the Ph.Eur. method. The illustration in Fig. 2 summarizes QC procedure according to the SPC version valid at the time of the study (Package insert for Technescan MAG3. Mallinckrodt Suisse SA, 2003). Meanwhile, a new version of the SPC has been approved in 2015 (Package insert for Technescan MAG3. Mallinckrodt Suisse SA, 2015). The method has been simplified to two different elutions. Unfortunately, there is no indication of how to attribute the individual fractions to the two eluates described in the SPC. Possible impurities are characterized as hydrophiles (3%) and lipophiles (4%). We can only assume that eluate 1 and the SEP-PAK C18 cartridge contain the lipo- or hydrophile impurities, and that eluate 2 contains the ^99m^Tc-MAG3 (94%), as well as ^99m^Tc-MAG2. ^99m^Tc-MAG2 is not listed as possible impurity by the company. In the SPC version from 2003 (Package insert for Technescan MAG3. Mallinckrodt Suisse SA, 2003), the hydrophilic, lipophilic impurities and ^99m^Tc-MAG3 were individually eluted, measured and clearly attributed to the different eluates. Therefore, applying the SPC method of 2003 (Package insert for Technescan MAG3. Mallinckrodt Suisse SA, 2003) for our study seems to be more adequate to guarantee reasonable comparison to other methods as the attribution of the eluates is clearly described.

### New TCL method

The new two-strip TLC method for the routine QC of Mallinckrodt Pharmaceutical ^99m^Tc-MAG3 labeling we developed is based on a first separation on an iTLC-SG chromatography paper with a 60:40 % mixture of ethyl acetate and methyl ethyl ketone (MEK), already mentioned in the literature (Chen et al., 1993), combined with a new solvent for the second separation on the same type of support, with a 90:10 % ethanol/water mixture.

Two iTLC-SG strips, each 2 - 2.5 cm wide and 10 cm long, were marked with a pencil at 1.5 cm from the bottom of the strip (“start”) and a second line at 8.5 cm indicating the solvent front (“front”). The strips were dried at 100°C for 1 hour and stored in a desiccator with silica gel. The conditioned strips were removed from the desiccator only prior to use (max. 30 minutes). For the chromatography strip one, a solvent mixture of 6 ml ethyl actetate and 4 ml methyl ethyl ketone (EtAc/MEK, 60:40) was prepared and transferred to the chromatography tank. For strip two, 9 ml of ethanol and 1 ml of distilled water (ETOH/H2O, ratio 90:10) were prepared the same way. The tanks were left to equilibrate for 10-15 minutes. A QC sample aliquot was applied on each strip and strips were developed in the corresponding tanks in parallel, until the solvent front. Strip 1 takes about 5 minutes to develop, strip 2 about 15 minutes. When finished, strips were immediately dried at room temperature before reading with a TLC scanner or measuring in a dose calibrator (TLC integration limit and dose calibrator cutting point at 6 cm from the bottom of the strip).

The RCP was calculated applying the following formula:

$$ \boldsymbol{RCP}\left(\%\right)=100\%-\left(\% impurities\ strip\ 1+\% impurities\ strip\ 2\right) $$

Where:

$$ \% impurities\ strip\ 1=\frac{activity\  top\  part\ x\ 100}{activity\  top+ bottom\ \mathrm{part}}=99 mTcO4+ TcMAG2 $$

$$ \% impurities\ strip\ 2=\frac{ activity\ bottom\ part\ x\ 100}{activity\  top+ bottom\ part}=(TcO2)n+ Tc Tartrate+ Tc(MAG3)x $$

### Validation methodology

The results of the new quality control method were validated comparing to HPLC and PC measurements referring to specifications of the official method in force from the company (Package insert for Technescan MAG3. Mallinckrodt Suisse SA, 2015) and the Ph.Eur method (European Pharmacopoeia Edition 7.0, 2008). Validation was performed following the ICH validation guidelines (International Commission on Harmonisation, 1996). We tested for specificity, accuracy, limit of detection (LoD), robustness and precision.

### Specificity

The specificity of the analytical method was tested to sufficiently separate the desired substance from its possible impurities. We tested specificity for free pertechnetate (^99m^TcO_4_), colloidal ((^99m^TcO_2_)_n_), ^99m^Tc-tartrate and ^99m^Tc-Mag2 (e.g. (Bormann et al., 1995; van Hemert et al., 2005; Nosco et al., 1993; Seetharaman et al., 2006; Chen et al., 1993)). The first three impurities are considered as hydrophilic and ^99m^Tc-Mag2 as a lipophilic impurity. To test the migration behavior and occurrence of these impurities, the following reference solutions have been prepared and measured:

Solution 1 (^99m^TcO_4_): Saline eluate from generator (approx. 250 MBq/ml); Solution 2 ((^99m^TcO_2_)_n_): 0.025 ml of SnCl_2_ solution (1.7 mg/ml) were added to ca. 500 MBq ^99m^TcO_4_ and let to equilibrate (gentle shaking); Solution 3 (^99m^Tc-Tartrate): 0.5 ml of Na-K tartrate solution (0.4 g dissolved in 10 ml of highly purified water), 0.025 ml of tin chloride solution (1.7 mg/ml) and ca. 500 MBq ^99m^TcO_4_ were added and let to equilibrate (gentle shaking). The quantitative formation of ^99m^Tc-tartrate was confirmed by HPLC analysis; Solution 4 (^99m^Tc-MAG2): 5 mg of S-benzyl-Mag2 (synthesized employing method for ^99m^Tc-MAG3 as described in Brandau et al. (Brandau et al., 1988)), were dissolved in 5 ml of 1-mM-NaOH at 80 °C for 5 min. A septum vial was purged with nitrogen gas and 1 ml of MAG2 solution and 1ml of Na-K tartrate solution, 0.025 ml of SnCl_2_ solution and ca. 500 MBq ^99m^TcO_4_ were added. The mixture was heated at 100 °C for 10 minutes and allowed to cool at room temperature. The quantitative formation of ^99m^Tc-Mag2 was confirmed by HPLC analysis.

### Accuracy

The accuracy was validated by comparing RCP values of our two-strip method with the Ph.Eur. method (European Pharmacopoeia Edition 7.0, 2008) for the same preparation of ^99m^Tc-MAG3.

### Limit of detection and quantification of impurity limit

The limit of detection was tested by performing linearity tests on our instruments. A deposited activity of about 1MBq/10 μl was considered as reference value for the quantification of impurities (Package insert for Technescan MAG3. Mallinckrodt Suisse SA, 2003). Based on the criteria set in the SPC (Package insert for Technescan MAG3. Mallinckrodt Suisse SA, 2003; Package insert for Technescan MAG3. Mallinckrodt Suisse SA, 2015) and the ones defined in the Ph.Eur. monograph (European Pharmacopoeia Edition 7.0, 2008), we decided setting the quantitative impurity limit to 3 % impurities per TLC strip to guarantee a RPC of minimum 94%.

### Robustness

To test the robustness different spot volumes (QC sample aliquot) and variations in proportions of the solvent mixtures were applied. Additionally, different batches of iTLC-SG papers were employed.

### Precision: Repeatability and intermediate precision

Repeatability was tested on three different labelings of the same batch production of ^99m^Tc-MAG3 applying identical conditions. Each labeling was measured at three different times, making three chromatographic separations. An HPLC measurement was performed on the same batch. Intermediate precision was tested with different laboratories, operators, equipment and days.

## Results

### SPE Method of the company

As already revealed by other studies (Chen et al., 1993; Murray et al., 2000; Vinberg, 2000; Ponto, 2005; Millar & Hesslewood, 2004), the SPE method for RPC testing suggested by the company (Package insert for Technescan MAG3. Mallinckrodt Suisse SA, 2003) shows varying results and seems to be depending on the elution rate. Our results confirm an inverse correlation between radiochemical purity and elution rates: the higher the elution rate, the lower the RCP (table 3 and 4). Elution rates ≥10ml/min result in RCP values of <95%. Applied elution rates of 1.7, 10, 20 and 30 ml/min (Table 3) compared with Ph.Eur. method (table 4, three analyses done at approximately the same time points as the SEP-PAK measurements) indicate an increasing trend of hydrophilic and of lipophilic components with elution rates ≥10ml/min.

**Table 3:** Table showing the RCP of ^99m^Tc-MAG3 with the SPE-method, resulting from different elution rates applied. The cartridge was also measured (not requested in SPC 2003) for quantification of (^99m^TcO_2_)_n_ which is retained on the cartridge

Elution rate	Hydrophilic compounds	^99m^Tc- MAG3	Lipophilic compounds	(^99m^TcO_2_)_n_(cartridge)
[ml/min]	[%]	[%]	[%]	[%]
1.7	2.0	95.8	1.7	0.5
10	4.1	93.8	1.8	0.4
10	3.9	93.3	2.4	0.4
20	8.6	86.6	4.3	0.5
30	8.4	79.8	11.3	0.6

**Table 4:** Results of corresponding HPLC measurements at 3 different time points in comparison with SPE- method results from table 3. The SPC in force today does not mentioned which impurities are attributed to the individually eluted fractions on the SEP-PAK columns and the (^99m^TcO_2_)_n_ is not taken into account (not measured).

	Hydrophilic compounds	^99m^Tc- MAG3	Lipophilic compounds
	[%]	[%]	[%]
HPLC 1	1.5	97.2	1.3
HPLC 2	0.6	98.0	1.4
HPLC 3	1.1	96.9	2.0

### New two-strip TLC method

The individual results of the validation criteria and comparison with the method of the Ph.Eur. (HPLC and PC data) are presented below (the complete validation data can be found in the tables in the Online Resource 1). The results are expressed with 2 standard deviations (k = 2) meaning that 95.4% of the results are in the mean values.

### Specificity

**Fig. 3:**
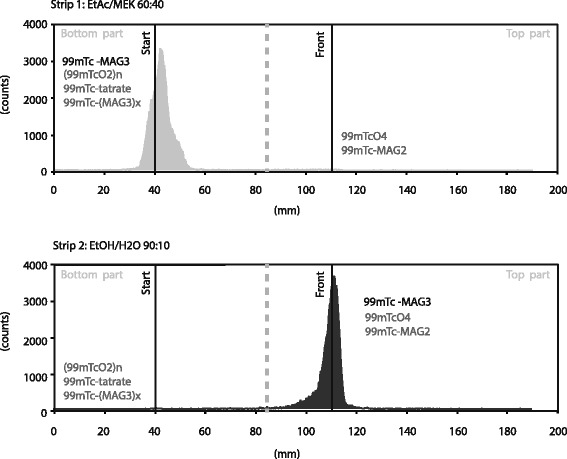
Illustration of the new two-strip TLC method. On strip one, the ^99m^TcO_4_ and ^99m^Tc-MAG2 migrate to the front, all other components stay at the origin. On strip two, 99mTc-MAG3, ^99m^TcO_4_ and ^99m^Tc-MAG2 migrate to the front; the other components stay at the origin (positioned at 40 mm on the TLC scanner)

The specificity was validated with samples of ^99m^Tc-MAG3 preparations and individual reference solutions of the main impurities, with the exception of pre-complexes, which are not available as isolated compounds. They are attributed to strip 2. Figure 3 shows an illustration of each TLC strip. ^99m^Tc-MAG3 stays at the origin for strip 1 and migrates to the solvent front for strip 2 similar as in the method of Chen et al. (Chen et al., 1993). Colloidal (^99m^TcO_2_)_n_ stays at the origin with both solvents. ^99m^Tc-tartrate stays in the lower part of both strips and is therefore separated from the ^99m^Tc-MAG3 on strip 2. ^99m^TcO_4_ and ^99m^Tc-MAG2 migrate to the front of both strips and can be quantified together as impurities on the upper part of strip 1.

### Accuracy

On three consecutive days one batch of ^99m^Tc-MAG3 was prepared and analyzed with the new TLC and the Ph.Eur. method. Three measurements were done each time and quantified with the TLC scanner and the dose calibrator. For the three replicates, three different batches of iTLC sheets were used as part of the robustness validation. No significant differences were observed. An HPLC measurement was performed on each batch and taken as the reference value for accuracy identification. Results for accuracy are summarized in table 5.

**Table 5:** Comparison of RCP values determined by the new TLC method by scanner or dose calibrator against the RCP obtained by the Ph.Eur. method (HPLC and PC) taken as reference (standard deviation k=2)

		Ph.Eur.	2-strip method				Accuracy	
Date	Validation	(HPLC&PC)	TLC Scanner		Dose calibrator		Difference to Ph.Eur. method	
		[%]	[%]		[%]		TLC Scanner	Dose calibrator
							[%]	[%]
27.04.2016	No 1	97	96.7	± 0.8	97.1	± 0.8	-0.3	0.1
28.04.2016	No 2	96.9	96.7	± 0.5	97.1	± 0.8	-0.2	0.2
29.04.2016	No 3	95.8	96.1	± 0.4	Not measured		0.3	N.A.
29.04.2016	No 4		95.6	± 0.5	96.1	± 0.5	-0.2	0.3

### Limit of detection and quantification of impurity limit

A linearity of the dose calibrators and the TLC scanners with low ^99m^Tc activities was realized for each instrument. The detection limit obtained for our systems is 2 kBq for the TLC scanner and 10 kBq for the dose calibrator. The impurity limit being fixed at ≤ 3%, an activity of 30 kBq can therefore be quantified and is within the linear range of measurements.

### Robustness

Robustness was tested for proportion in solvent mixtures and deposited sample volume as well as different batches of iTLC-SG papers. The kit was heated to 70°C only to increase the occurring impurities. No variations based on mixtures were observed, similar as already observed in Chen et al. (Chen et al., 1993). Table 6 and 7 show the results of RCP in correlation with modified solvent mixtures for the two strips. We observe no significant influence on the obtained RCP.

**Table 6:** Results of impurities found applying deviations in solvent mixtures for strip 1

Sample	Strip 1: Ratio EtAc/MEK		
	55:45	60:40	65:35
	Impurities (%)	Impurities (%)	Impurities (%)
Sample 1	6.5	6.1	6.0
Sample 2	6.6	6.5	6.0
Sample 3	6.7	6.4	6.6
Average	6.6	6.3	6.2
STD *(k=2)*	0.2	0.4	0.6

**Table 7:** Results of impurities found applying deviations in solvent mixtures for strip 2

Sample	Strip 2: Ratio EtOH/H_2_O		
	87:13	90:10	93:7
	Impurities (%)	Impurities (%)	Impurities (%)
Sample 1	5.0	5.3	5.5
Sample 2	4.9	5.1	5.3
Sample 3	4.6	5.5	5.0
Average	4.8	5.3	5.3
STD *(k=2)*	0.4	0.4	0.6

**Fig. 4:**
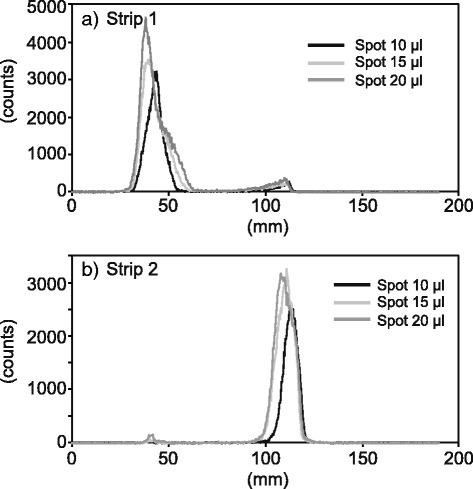
TLC chromatograms with QC samples applied of 15, 15 and 20 microliters

Figure 4 shows the chromatograms for different volumes applied. A small influence of differences in volume of the QC sample was observed mainly on strip 1. The front peak of the ^99m^Tc-MAG3 is larger with a bigger sample volume but does still not influence its interpretation. Volumes of 10, 15, and 20 μl were tested and did not reveal significant variation in RCP.

The tests using three different batches of iTLC papers (production years 2013, 2015 and 2016) did not reveal any significant difference in RCP (see Online Resource 1 for individual results).

*Intermediate precision*

Different operators, TLC scanners, dose calibrators or days did not significantly influence the RCP results. We observe a good correlation between references values (Ph.Eur. method by HPLC and TLC (European Pharmacopoeia Edition 7.0, 2008)) and our two-strip TLC method (see table 5 and validation data in Online Resource 1).

## Discussion

The main goal of our study was to develop a simple and fast TLC method identifiying the main impurities occurring in the heated formulation of a MAG3 preparation (Package insert for Technescan MAG3. Mallinckrodt Suisse SA, 2003; Package insert for Technescan MAG3. Mallinckrodt Suisse SA, 2015). Specifications should at minimum meet the criteria set by the company method in force, and additionally show good agreement to the Ph.Eur. method. Our review of official methods suggested for this kit preparation within different countries (US, EU and Switzerland) had revealed important differences between the methods applied for RCP testing. In table 1 and 2 we had summarized the official methods of RCP testing for heated and non-heated formulations and compared them to the Ph.Eur. method. We found that specifications for RCP vary between countries and one of the main impurities, colloidal (^99m^TcO_2_)_n_ is often not measured. Surprisingly, none of the suggested methods is fully compliant with the Ph.Eur. method. Testing our TLC method with other formulations of MAG3 (non-heated formulation), we found very good agreement between our RCP results and RCP based on the SPC method (Package insert for ROTOP-MAG-3 Kit. Heider AG, 2006). In comparison to the Ph.Eur. so far unsolved discrepancies remained for the non-heated formulation.

### SPE method

Our tests of the manufacturer SPE method in force with the SEP-PAK cartridges (Package insert for Technescan MAG3. Mallinckrodt Suisse SA, 2003) show that results don't comply reliably with the reference method of the Ph.Eur. (European Pharmacopoeia Edition 7.0, 2008). Specifications for RCP are not compliant with the Ph.Eur. monograph and the robustness of the method is not optimal due to operator dependency in elution velocity. Vinberg (Vinberg, 2000) reported 1.5 ml/min as maximum flow rate to achieve reliable results with the SPE method, but he did not present data on higher flow rates. Such a low flow rate is difficult to achieve by manual manipulation and it prolongs the procedure substantially; therefore, we studied higher flow rates. Indeed, we found that elution rates ≥10 ml/min applied to the kit did significantly over estimate the hydrophilic impurities in the preparation and some of the lipophilic components were wrongly attributed to the ^99m^Tc-MAG3 peak (table 3 and 4). Additionally, different to Murray et al. (Murray et al., 2000), we found that not only the hydrophilic, but also lipohilic impurities may be over estimated. We conclude that only an automated elution would guarantee a consistent application of the right elution rate; done manually, results risk varying importantly and possible radiochemical impurities cannot be identified satisfyingly. Incomplete information about elution rate and specification of the eluates in the SPC make this QC method questionable. Interestingly, its former version, which is the official version in force in European countries (Package insert for Technescan MAG3, 2015), had included these information. The reasoning to remove this information in the current version in Switzerland is not clear to us. In addition, the manipulation of the SEP-PAK columns for performing the QC does not comply with good radiation safety for the personnel. Due to relatively long handling time of the radioactive material and close contact to extremities, a higher irradiation may occur applying the SPE method. The missing information about the attribution of impurities as well the risk of extremity irradiation make this QC method rather unattractive and explain why it is not widely applied in the centers.

### New TLC method

The main radiochemical impurities of the ^99m^Tc-MAG3 preparations are identified by our two-strip TLC method, with the possible exception of pre-complexes, which seem distributed over a wider range on strip 2 resulting in some uncertainty. Nonetheless, the good correlation between the RCP determination by the Ph.Eur. method (European Pharmacopoeia Edition 7.0, 2008) and our method indicates that the pre-complexes are well taken into account in TLC strip 2 and individual identification is not necessary for guaranteeing accuracy of this method (table 5). Different to other studies (Seetharaman et al., 2006; Chen et al., 1993), our specificity test allowed us to confirm ^99m^Tc-MAG_2_ as a possible impurity in the ^99m^Tc-MAG3 formulation. In addition, our method permits separation of ^99m^Tc-MAG3 from ^99m^Tc-tartrate.

To be compliant with the Ph.Eur. method, we decided to set the impurity limits for hydrophilic and lipophilic impurities to 3%. Hydrophilic impurities are ^99m^Tc-precomplexes, pertechnetate (^99m^TcO_4_), colloidal form (^99m^TcO_2_)_n_ and ^99m^Tc-tartrate. The limit of 3% is justified as results of HPLC measurements (obtained by the Ph.Eur. method) at different temperature conditions had shown that (^99m^TcO_2_)_n_ shows relatively constant values (always below 1%). The main variation occurs in the ^99m^Tc-precomplexes, ^99m^TcO_4_ and ^99m^Tc-tartrate, the other hydrophilic impurities. Setting the limit to 3% for the sum of the impurities on strip 2 (^99m^Tc-precomplexes^, 99m^Tc-tartrate and (^99m^TcO_2_)_n_) enables us to respect the 2% for the colloidal form (^99m^TcO_2_)_n_. If we would have more than 2% (^99m^TcO_2_)_n_, the other hydrophilic impurities, are likely to be increased. To set a total limit of 3% seems therefore justified.

The results of our study confirm the ability of our systems to measure the limit values of impurities in the case of the chosen activities deposited on a chromatography paper. The used instruments fulfill our acceptance criteria set (30 kBq) for quantification of the impurities. In the case of a very low specific activity, the dose calibrator detection may be used with precaution and the use of a gamma counter or TLC scanner is recommended. This is not specific to our method, but to any quality control method of radiopharmaceuticals prepared with low specific activities. Differences in volume of sample for TLC chromatograms may modify the separation between the compounds. Too large volumes can widen the peaks and reduce their resolution. The presence of a large amount of water (the main medium of a ^99m^Tc-MAG3 labeling) may also interfere with a separation. In our method, the influence of differences in volume is more important for strip 1 where the majority of the sample is not migrating. The deposited volume widens the base of the ^99m^Tc-MAG3 peak but no influence on RCP interpretation is observed as the peaks are well separated and there is no risk of wrong attribution of components (fig. 4). Our results show no significant variations in RCP within modified solvents mixtures (tables 6 and 7). Generally, we recommend the use of a micro pipette for the addition of solvents to prevent important variations in volumes.

## Conclusion

SPC methods for RCP testing for the two existing formulations of 99mTc-MAG3, heated (Package insert for Technescan MAG3. Mallinckrodt Suisse SA, 2003; Package insert for Technescan MAG3. Mallinckrodt Suisse SA, 2015) and non-heated (Package insert for ROTOP-MAG-3 Kit. Heider AG, 2006) differ and results are not in accordance with the Ph.Eur. specifications. Our new two-strip TLC method, combining two separations on the same type of support with two different solvent mixtures, has been found to be adequate for the determination of the RCP of ^99m^Tc-MAG3 (Package insert for Technescan MAG3. Mallinckrodt Suisse SA, 2003). The RCP obtained by TLC does not deviate significantly from the reference RCP determined by HPLC and PC following the Ph.Eur. method (European Pharmacopoeia Edition 7.0, 2008). The limit value for the total RCP is identic. This was not the case in former proposed methods where some impurities have been excluded and declared as insignificant (Seetharaman et al., 2006; Chen et al., 1993; Package insert for Technescan MAG3. Mallinckrodt Suisse SA, 2003; Package insert for ROTOP-MAG-3 Kit. Heider AG, 2006; Package insert for Technescan MAG3. Mallinckrodt Suisse SA, 2015; Package insert for Technescan MAG3, 2015; Package insert for Technescan MAG3, 2015; Package insert for ROTOP-MAG-3 Kit, n.d.-a; Package insert for ROTOP-MAG-3 Kit, n.d.-b). The specificity and accuracy of our method have been confirmed and results of total RCP are in very good agreement with the Ph.Eur. reference method. The limit of detection meets the limit test criteria necessary to identify the total amount of impurities in a TLC QC method. Its precision and robustness have been successfully validated. Results of the intermediate fidelity show a good correlation with the Ph.Eur. reference values, both for the TLC-scanner and the dose calibrator measurements. This two-strip TLC method could therefore be established for routine quality control of ^99m^Tc-MAG3 kits by introducing it into the SPC of the products (Package insert for Technescan MAG3. Mallinckrodt Suisse SA, 2003; Package insert for ROTOP-MAG-3 Kit. Heider AG, 2006; Package insert for Technescan MAG3. Mallinckrodt Suisse SA, 2015).

## Additional file

Additional file 1: ESM_1. (XLSX 47 kb)

## Abbreviations

EtAc: Ethyl acetate
ETOH: Ethanol
HPLC: High-pressure liquid chromatography
MEK: Methyl ethyl ketone
PC: Paper chromatography
Ph.Eur.: European Pharmacopoeia
QC: Quality control
RCP: Radiochemical purity
SPC: Summary of product characteristics
SPE: Solid-phase extraction
TLC: Thin layer chromatography
